# Mortality and Morbidity Among Nursing Home Residents Exposed to Hurricane Irma

**DOI:** 10.1093/geroni/igaa057.2608

**Published:** 2020-12-16

**Authors:** David Dosa, Julianne Skarha, Lindsay Peterson, Dylan Jester, Nazmus Sakib, Jessica Ogarek, Ross Andel, Kathryn Hyer

**Affiliations:** 1 Brown University, Barrington, Rhode Island, United States; 2 Brown University, Providence, Rhode Island, United States; 3 University of South Florida, Tampa, Florida, United States; 4 Brown University, PROVIDENCE, Rhode Island, United States

## Abstract

We combined Medicare claims and nursing home (NH) administrative data to determine the mortality and morbidity effect of Hurricane Irma on nursing home residents. We utilized the Centers for Medicare and Medicaid Services (CMS) Standard Analytical Files (SAFs) combined with the Minimum Data Set (MDS) to create an exposure cohort of NH residents residing in Florida facilities immediately prior to Hurricane Irma’s landfall on September 10, 2017. We created a control group of residents who resided in the same NHs over the same dates in 2015, a year when there were no hurricanes. Outcome variables included 30/90-day mortality and first hospitalizations post storm. Compared to the control, an additional 260 more NH deaths were identified at 30 days and 429 more deaths at 90 days. Long stay residents (≥100 days) were at particular risk for mortality compared to short stay residents (<100 days). Hospitalization was also markedly increased.

